# Stratified assessment of an FDA-cleared deep learning algorithm for automated detection and contouring of metastatic brain tumors in stereotactic radiosurgery

**DOI:** 10.1186/s13014-023-02246-z

**Published:** 2023-04-04

**Authors:** Jen-Yeu Wang, Vera Qu, Caressa Hui, Navjot Sandhu, Maria G. Mendoza, Neil Panjwani, Yu-Cheng Chang, Chih-Hung Liang, Jen-Tang Lu, Lei Wang, Nataliya Kovalchuk, Michael F. Gensheimer, Scott G. Soltys, Erqi L. Pollom

**Affiliations:** 1 Department of Radiation Oncology, Stanford University School of Medicine, 875 Blake Wilbur Drive, Stanford, CA 94305 USA; 2 Vysioneer Inc, Cambridge, MA USA

## Abstract

**Purpose:**

Artificial intelligence-based tools can be leveraged to improve detection and segmentation of brain metastases for stereotactic radiosurgery (SRS). VBrain by Vysioneer Inc. is a deep learning algorithm with recent FDA clearance to assist in brain tumor contouring. We aimed to assess the performance of this tool by various demographic and clinical characteristics among patients with brain metastases treated with SRS.

**Materials and methods:**

We randomly selected 100 patients with brain metastases who underwent initial SRS on the CyberKnife from 2017 to 2020 at a single institution. Cases with resection cavities were excluded from the analysis. Computed tomography (CT) and axial T1-weighted post-contrast magnetic resonance (MR) image data were extracted for each patient and uploaded to VBrain. A brain metastasis was considered “detected” when the VBrain- “predicted” contours overlapped with the corresponding physician contours (“ground-truth” contours). We evaluated performance of VBrain against ground-truth contours using the following metrics: lesion-wise Dice similarity coefficient (DSC), lesion-wise average Hausdorff distance (AVD), false positive count (FP), and lesion-wise sensitivity (%). Kruskal–Wallis tests were performed to assess the relationships between patient characteristics including sex, race, primary histology, age, and size and number of brain metastases, and performance metrics such as DSC, AVD, FP, and sensitivity.

**Results:**

We analyzed 100 patients with 435 intact brain metastases treated with SRS. Our cohort consisted of patients with a median number of 2 brain metastases (range: 1 to 52), median age of 69 (range: 19 to 91), and 50% male and 50% female patients. The primary site breakdown was 56% lung, 10% melanoma, 9% breast, 8% gynecological, 5% renal, 4% gastrointestinal, 2% sarcoma, and 6% other, while the race breakdown was 60% White, 18% Asian, 3% Black/African American, 2% Native Hawaiian or other Pacific Islander, and 17% other/unknown/not reported. The median tumor size was 0.112 c.c. (range: 0.010–26.475 c.c.). We found mean lesion-wise DSC to be 0.723, mean lesion-wise AVD to be 7.34% of lesion size (0.704 mm), mean FP count to be 0.72 tumors per case, and lesion-wise sensitivity to be 89.30% for all lesions. Moreover, mean sensitivity was found to be 99.07%, 97.59%, and 96.23% for lesions with diameter equal to and greater than 10 mm, 7.5 mm, and 5 mm, respectively. No other significant differences in performance metrics were observed across demographic or clinical characteristic groups.

**Conclusion:**

In this study, a commercial deep learning algorithm showed promising results in segmenting brain metastases, with 96.23% sensitivity for metastases with diameters of 5 mm or higher. As the software is an assistive AI, future work of VBrain integration into the clinical workflow can provide further clinical and research insights.

## Introduction

Brain metastases are the most common central nervous system malignancy and affect up to 30–40% of cancer patients [1]. Stereotactic radiosurgery (SRS) is an accepted standard of care for the treatment of limited brain metastases (Brown et al. 2016). Two critical steps in planning for SRS are the identification and localization of individual brain metastases on the patient scans and the delineation of the tumor boundaries by the radiation oncologist and/or neurosurgeon. The latter process can be time-consuming and subject to a high degree of inter-observer variability, especially for small brain metastases [2–4].

Artificial intelligence (AI) has demonstrated promise in addressing these issues. With the goal of improving efficiency and standardization, machine learning models have recently been developed for automated detection and segmentation of metastatic brain tumors [2, 5–12]. However, the published literature thus far is comprised of technical proof-of-concepts in which the model is tested on small, limited sample sizes, and/or it is not readily deployable to the clinic.

VBrain is a deep learning (DL) algorithm patented by Vysioneer Inc. that received medical device clearance by the Food and Drug Administration (FDA) in 2021 and has been shown to significantly improve inter-reader agreement, contouring accuracy, and efficiency [13, 14]. We aim here to validate this tool in a heterogenous cohort of patients who have been treated with SRS for brain metastases at a single institution as well as provide guidance for the scope of its use.

## Methods

### Retrospective patient cohort

We obtained approval from Stanford University institutional research ethics board to conduct this study. Our institution has extensive experience with SRS of brain metastases, as previously described [15]. We included 100 randomly selected patients with unresected brain metastases treated with SRS at our institution from 2017 to 2020. Patients who had prior intracranial resection or intracranial radiation were excluded.

### Deep learning-based algorithm

VBrain is a commercial, FDA-cleared DL-based algorithm that uses magnetic resonance imaging (MRI) and computed tomography (CT) to segment brain metastases. VBrain adopts the ensemble strategy to optimize the segmentation results: 3D U-Net addresses overall tumor segmentation with high specificity while the DeepMedic model focuses on smaller lesions with a high sensitivity [14–16]. The network was trained with a novel volume-aware Dice loss function, which uses information about lesion size to enhance the sensitivity of small lesions [17].

### Workflow for automatic detection and segmentation

For each patient, three sets of Digital Imaging and Communications in Medicine (DICOM) files used during SRS planning were exported from our institutional CyberKnife and/or Picture Archiving and Communication System: (1) the CT scan, (2) the axial T1-weighted post-contrast fast spoiled gradient echo MR scan, and (3) the Radiotherapy Structure Set (RTSS). The files were stripped of the protected health information contained in the DICOM headers using a custom script and relabeled using a unique study ID. The anonymized CT and MR scans for each patient were processed by the VBrain software to generate an RTSS with automatically identified and contoured brain metastases.

### Evaluation

Subsequent analyses compared the two RTSSs: output contours from VBrain against the physician-defined contours used for SRS. A brain metastasis was considered “detected” when the VBrain- “predicted” contours overlapped with the corresponding physician contours (“ground-truth” contours). We evaluated performance of the predicted contours against ground-truth contours using the following metrics: lesion-wise Dice similarity coefficient (DSC), lesion-wise average Hausdorff distance (AVD), false positive (FP) count, and lesion-wise sensitivity (%).

The lesion-wise DSC was evaluated for only detected lesions, defined as ground-truth lesions that contained within them the centroid of a predicted lesion. FP was defined as the predicted regions which do not overlap with any ground-truth lesion. Lesion-wise sensitivity was defined as the ratio of the total number of detected lesions by VBrain to the total number of ground-truth lesions. Due to the small tumor sizes of the cohort, we also reported the lesion-wise sensitivities with effective diameters equal to and greater than 10 mm, 7.5 mm, and 5 mm, where the effective diameter was defined as the diameter of a volume-equivalent sphere.

The patient cohort was stratified by demographics (age, sex, race) and clinical characteristics (histology type, lesion count, and lesion size) to identify whether significant differences in performance existed in certain groups. Kruskal–Wallis tests were performed to assess the relationships between patient characteristics (including sex, race, histology type, age, and size and number of brain metastases) and performance metrics (including mean lesion-wise DSC, mean lesion-wise AVD, mean FP count, and lesion-wise sensitivity). All tests used a significant p-value threshold of 0.05 unless stated otherwise. All statistical analyses were conducted using the SciPy v1.5.2 package in Python 3.8.7.

## Results

### Patient demographics

We analyzed 100 patients with 435 intact brain metastases treated with SRS at our institution. Demographic characteristics for our patient cohort are summarized in Table 1. The median number of brain metastases per patient was 2 (range: 1 to 52), and the median tumor size was 0.112 c.c. The most common primary histologies were lung (56%), melanoma (10%), and breast (9%).

**Table 1:** Demographic and clinical cohort characteristics consisting of 435 brain metastases distributed across 100 patients

Characteristic	N
Age (median, years)	69 (range 19–91)
*Sex*	
Female	50
Male	50
*Race*	
White	60
Asian	18
Black/African American	3
Native Hawaiian/Pacific Islander	2
Other/unknown/not reported	17
*Ethnicity*	
Hispanic/Latino	88
Not Hispanic/Latino	11
Unknown/not reported	1
*Year of radiosurgery treatment*	
2017	35
2018	28
2019	26
2020	11
*Histology type*	
Non-small cell lung cancer	52
Melanoma	10
Breast	9
Gynecologic	8
Renal cell carcinoma	5
Gastrointestinal	4
Small cell lung cancer	4
Sarcoma	2
Other	6
*Number of brain metastases per patient*	
Median/patient	2 (range/patient 1–52)
1 metastasis	30
2 metastases	26
3 metastases	7
4 metastases	8
> = 5 metastases	29
*Tumor size in c.c*	
Mean	1.078 (range 0.010–26.475)
Q1	0.040
Q2	0.112
Q3	0.521
*Input CT scan*	
Median slice thickness in mm	1.250 (range 1.000–1.250)
Median pixel resolution in mm	0.546 (range 0.443–0.977)
*Input MR scan*	
Median slice thickness in mm	1.000 (range 0.500–1.200)
Median pixel resolution in mm	0.469 (range 0.469–0.938)

### Overall performance and stratified assessment

Comparison metrics evaluating performance of VBrain against clinical ground-truth contours for all brain metastases are described in Table 2. We found mean lesion-wise DSC to be 0.723, mean lesion-wise AVD to be 7.34% of lesion size (0.704 mm), mean FP count to be 0.72 tumors per case, and lesion-wise sensitivity to be 89.30% and 96.23% for all lesions and lesions with diameter greater than 5 mm. Furthermore, sensitivity was found to be 85.37% and 90.23% for patient cases with one or two metastases and with three or more metastases, respectively.

**Table 2:** Performance metrics

Performance metric	
Mean lesion-wise DSC	0.723
Mean lesion-wise AVD	7.34% (0.704 mm)
Mean FP count	0.72
Lesion-wise sensitivity	89.30%
Lesion-wise sensitivity (diameter > = 5 mm)	96.23%
Lesion-wise sensitivity (patient with tumor count < = 2)	85.37%
Lesion-wise sensitivity (patient with tumor count > = 3)	90.23%

Dice similarity coefficient (DSC), average Hausdorff distance (AVD), false positive (FP) count, and lesion-wise sensitivity (%) for our cohort of 435 metastases across 100 patients

As shown in Table 3, sensitivity was found to be 99.07%, 94.83%, and 93.94% for lesions with diameter equal to and greater than 10 mm, between 7.5 mm and 10 mm, and between 5 and 7.5 mm, respectively. The size of the brain metastases was significantly associated with lesion-wise DSC (*p* < 0.001) and sensitivity (*p* < 0.001), and the number of brain metastases per patient significantly correlated with sensitivity (*p* < 0.05; Table 4).

**Table 3:** Lesion-wise sensitivity by effective diameter of brain metastases

Sensitivity	%
Effective diameter > = 5 mm & < 7.5 mm	93.94
Effective diameter > = 7.5 mm & < 10 mm	94.83
Effective diameter > = 10 mm	99.07

**Table 4:** Kruskal–Wallis tests were performed to assess the relationships between patient and lesion characteristics, and performance metrics

Characteristics	*p*-Value			
	Lesion-wise DSC	Lesion-wise AVD [mm (%)]	FP Count	Sensitivity (%)
*Patient level*				
Sex	0.546	0.771 (0.726)	0.459	0.146
Age	0.118	0.899 (0.226)	0.838	0.566
Race	0.529	0.618 (0.418)	0.433	0.446
Primary histology	0.186	0.616 (0.160)	0.519	0.613
Tumor count	0.085	0.207 (0.858)	0.835	0.030
*Lesion level*				
Tumor size	1.64e−31	0.484 (2.54e−29)*	N/A**	5.05e−09

*AVD in terms of absolute distance (mm) is not correlated with tumor size. There is a correlation between relative AVD (%) and tumor size, because relative AVD decreases as tumor size increases

**Statistical result cannot be assessed because there is no ground-truth size for false positives

**Fig. 1:**
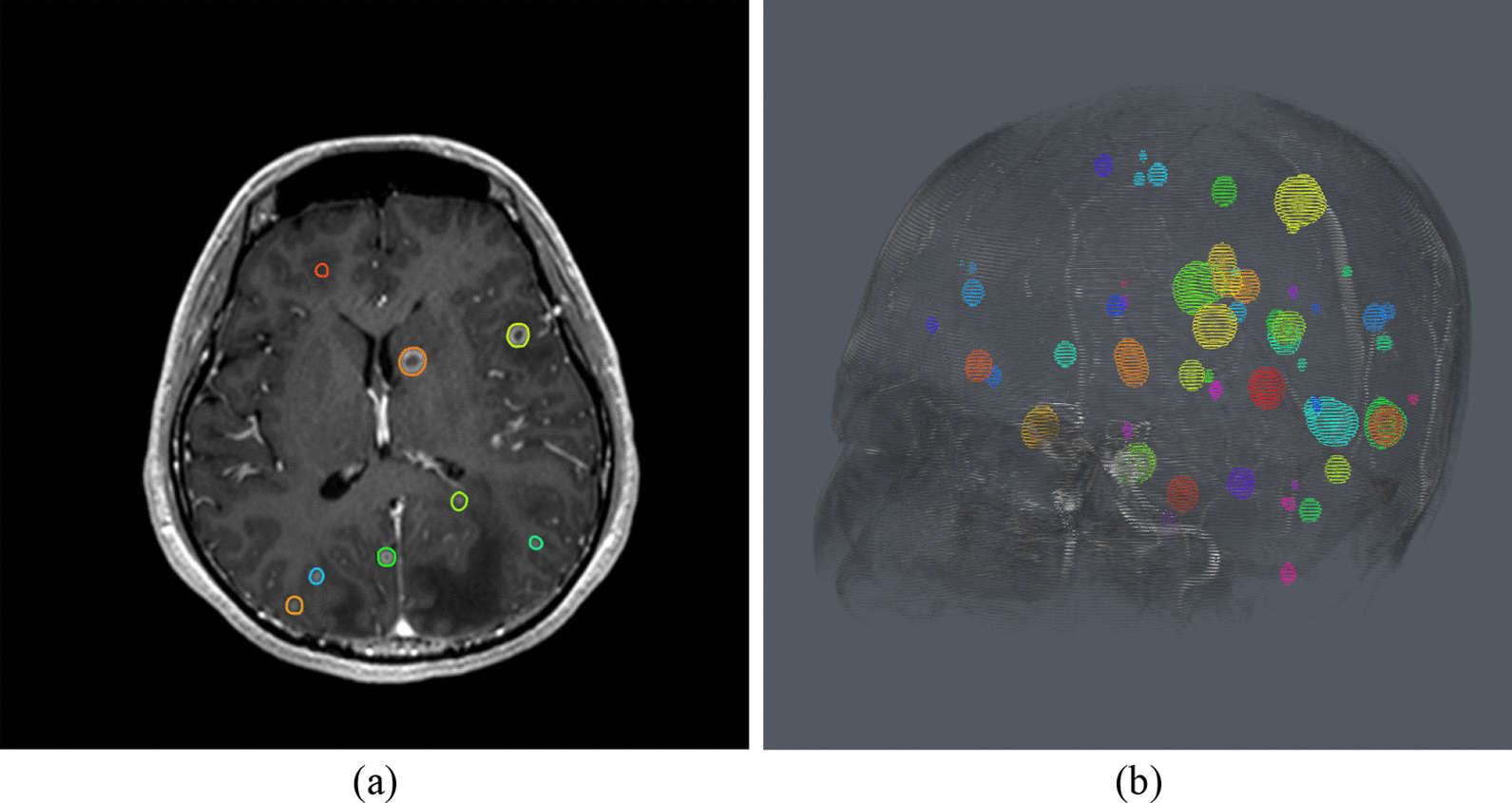
Case with 52 Lesions. **a** Axial view. **b** 3D view. VBrain successfully predicted multiple brain metastases for this patient case with over 50 brain metastases, as this case had a Dice similarity coefficient (DSC) of 0.813, average Hausdorff distance (AVD) of 3.81% (0.511 mm), false positive count (FP) of 0, and sensitivity (%) of 90% and 100% for overall and >  = 5 mm tumors, respectively

**Fig. 2:**
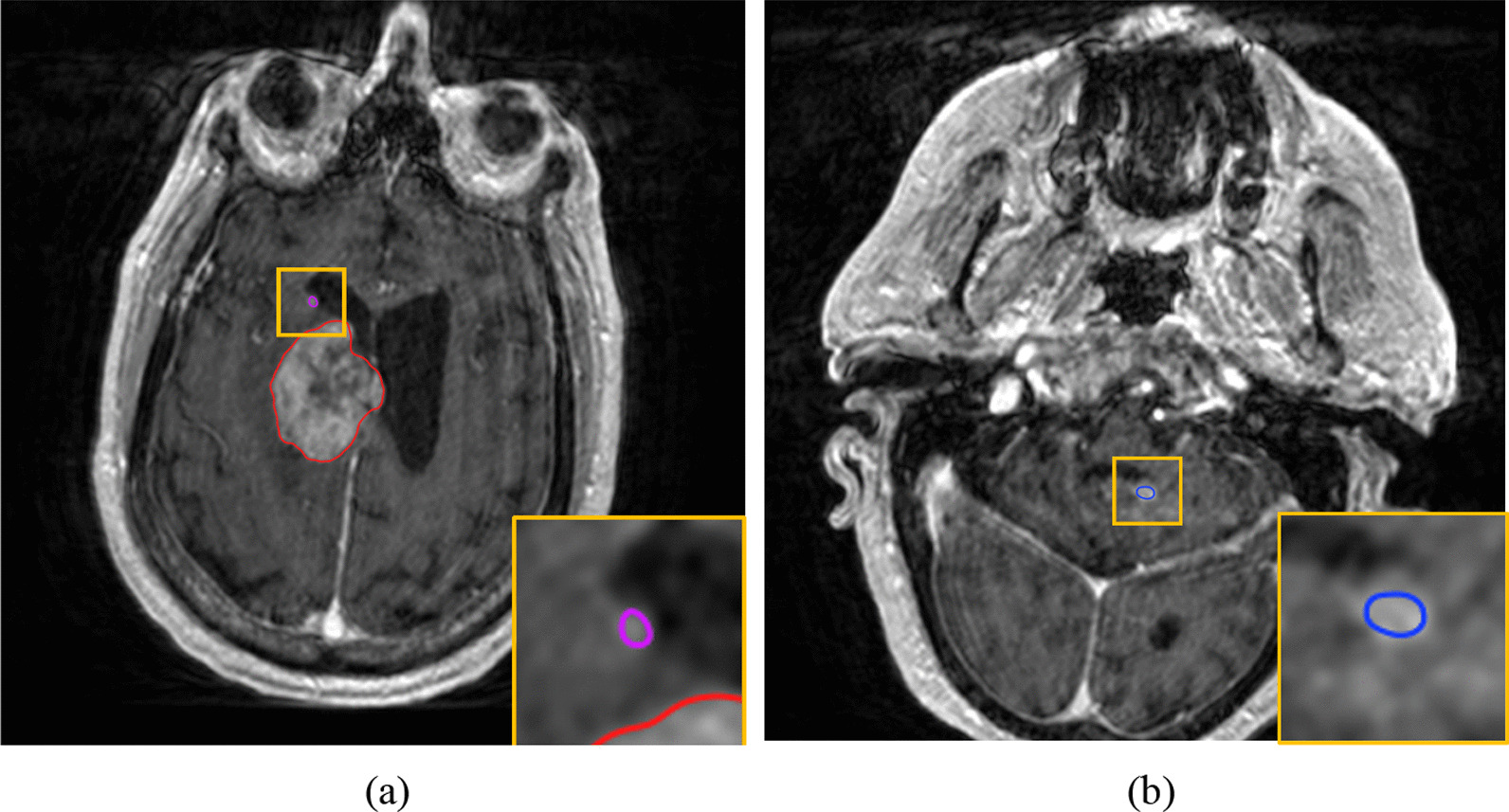
Case with Tiny Lesions. As highlighted by the bounding box, VBrain successfully contoured brain metastases with a diameter of 2.5 mm (**a**) and 4.2 mm (**b**). This case had a Dice similarity coefficient (DSC) of 0.944, average Hausdorff distance (AVD) of 1.78% (0.828 mm) false positive count (FP) of 1, and sensitivity (%) of 100% for both overall and >  = 5 mm tumors

**Fig. 3:**
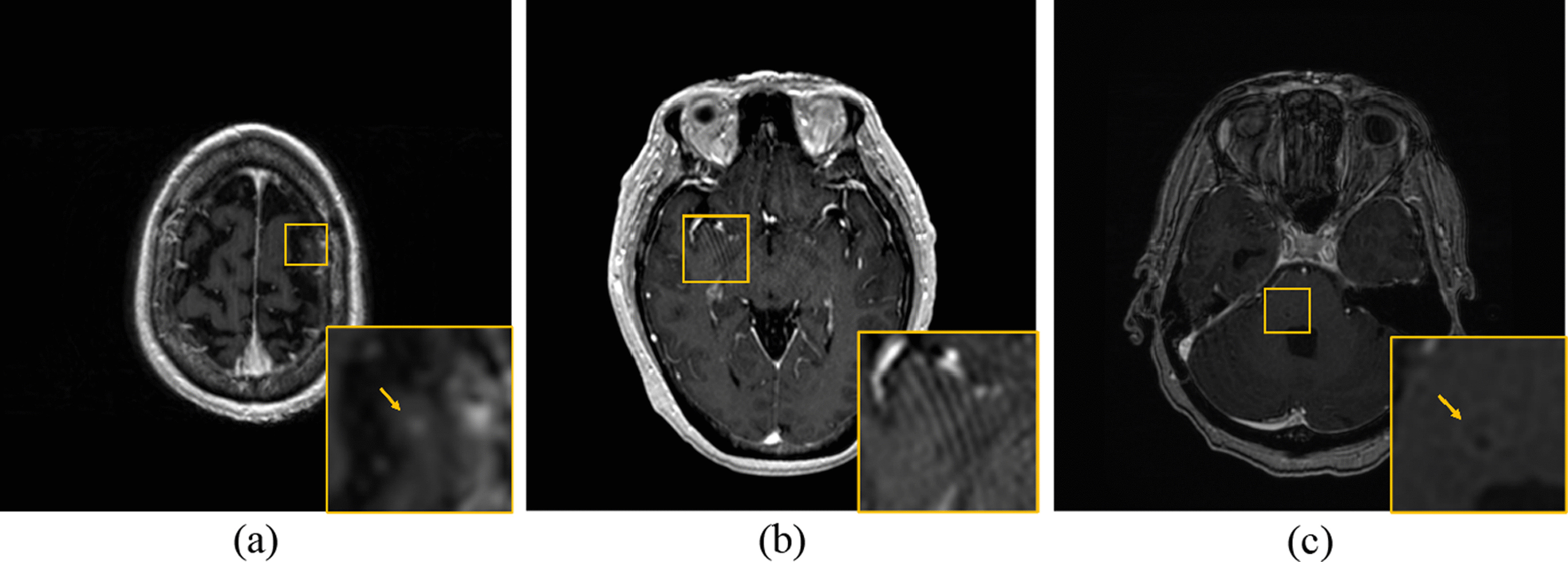
Challenging Cases: **a** Tiny Lesion. (0.02 c.c.) **b** Image Artifacts. **c** Insufficient Contrast. In these cases, the diagnostic report and longitudinal images may be required for additional reference

Figure 1 and Fig. 2 illustrate cases in which VBrain effectively predicted brain metastases among patients with numerous lesions (52) and lesions of small size (2.5 and 4.2 mm diameters). Figure 3 demonstrates challenging cases with tiny lesions, poor image quality, or insufficient contrast in the MR scan for which diagnostic reports and/or longitudinal images might be required for additional reference. No other statistically significant differences in performance metrics were observed across demographic and clinical characteristic groups.

## Discussion

Our analysis included 435 brain metastases in 100 randomly selected patients who were treated with SRS at our institution. This analysis contains significantly more brain metastases and smaller brain metastases than other published series evaluating brain metastases segmentation algorithms [6]. The median tumor size in our study was 0.112 c.c., which is 5–10 times smaller than other cohorts [9, 18]. Smaller lesions are more challenging to detect as well as segment [19]. However, increasingly smaller lesions are being treated with radiation now with improvements in imaging and treatment capabilities. Thus, it is critical to evaluate the performance of available auto-segmentation software for these lesions. Further, many of the previous papers used their cohorts to perform both training and validation. Our study used the entire cohort to perform external validation of VBrain. The primary cancer site distribution of our study cohort is representative of the general population with brain metastases, which includes mostly lung (40–50%), breast (15–25%), and skin (5–20%) primaries [20].

DSC and sensitivity were all found to be significantly associated with the size of brain metastases. 99.07% lesion-wise sensitivity was achieved for tumors greater than 10 mm but decreased to 97.59% and 96.23% for lesions greater than 7.5 mm and 5 mm. Furthermore, sensitivity was found to be significantly associated with tumor counts. There were no other significant associations between patient characteristics and VBrain performance metrics.

There are some limitations to this study. First, these patients were treated at a single academic institution with extensive radiosurgical experience and dealing with, on average, more and smaller brain metastases, which may limit generalizability. Smaller intracranial lesions are difficult to be identified and contoured, which is a common challenge with any segmentation method, manual or automated [19]. Thus, VBrain’s performance in this study may underestimate its overall performance on a general patient population. Second, we excluded patients with prior intracranial radiation or surgical resection. Although these patients represent a minority of radiosurgical cases, further work will be needed to evaluate VBrain’s ability to differentiate between resection cavities, pre-treated lesions, and untreated lesions. Finally, it is important to note that thin-slice 3 T MRI brain with contrast scans should generally be used for SRS contouring [21] but were available for 98% of the patient cases in this study.

VBrain is a clinic-ready and FDA-cleared AI software intended to assist trained medical professionals by providing initial brain metastases contours. In a prior reader study evaluating five brain metastases cases, VBrain assistance significantly improved inter-reader agreement, contouring accuracy, and efficiency, and clinicians were able to detect 12% more lesions than they would have without the software [14]. Although VBrain has been shown to identify brain metastases missed by physicians and reduce contouring time, based on its intended use cleared by the FDA, this tool cannot replace the expertise of the treating physician who must review and modify the final treatment contours.

Future avenues of exploration for VBrain and other tumor auto-segmentation tools are their powerful potential for research application. For example, these tools can enable instantaneous tracking of brain metastases over serial MRIs to evaluate response to novel treatments as well as inform real-time clinical decision making. As advances in imaging and treatment-delivery capabilities enable the detection and treatment of increasingly complex cases of brain metastases, future work is ongoing to develop and improve AI tools to assist in SRS treatment planning.

## Data Availability

The data used in this study are not publicly available due to patient health privacy restrictions. However, anonymized data may be available from the authors upon reasonable request.
